# Expression Profiling of Proliferation and Apoptotic Markers along the Adenoma-Carcinoma Sequence in Familial Adenomatous Polyposis Patients

**DOI:** 10.1155/2013/107534

**Published:** 2013-02-13

**Authors:** Jayson Wang, Nabil El-Masry, Ian Talbot, Ian Tomlinson, Malcolm R. Alison, Mona El-Bahrawy

**Affiliations:** ^1^Department of Histopathology, Imperial College London, Hammersmith Hospital, DuCane Road, London W12 0HS, UK; ^2^Department of Surgery, Faculty of Medicine, Alexandria University, Alexandria, Egypt; ^3^Department of Surgery, Charing Cross Hospital, London W6 8RF, UK; ^4^Department of Histopathology, St. Mark's Hospital, London HA1 3UJ, UK; ^5^Wellcome Trust Centre for Human Genetics, University of Oxford and NIHR Oxford Biomedical Research Centre, Oxford OX3 7BN, UK; ^6^Centre for Tumour Biology, Barts Cancer Institute, Queen Mary, University of London, London EC1M 6BQ, UK

## Abstract

*Introduction*. Familial adenomatous polyposis (FAP) patients have a germline mutation in the adenomatous polyposis coli (*APC*) gene. The APC protein interacts with beta-catenin, resulting in the activation of the Wnt signalling pathway. This results in alterations in cell proliferation and apoptosis. We investigated the expression of beta-catenin and related proliferation and apoptotic factors in FAP patients, exploring the expression along the adenoma-carcinoma sequence. *Methods*. The expression of beta-catenin, p53, bcl-2, cyclin-D1, caspase-3, CD10, and Ki-67 proteins was studied by immunohistochemistry in samples of colonic nonneoplastic mucosa (*n* = 71), adenomas (*n* = 152), and adenocarcinomas (*n* = 19) from each of the16 FAP patients. *Results*. The expression of beta-catenin, caspase-3, cyclin-D1, and Ki-67 was increased in both adenomas and carcinomas in FAP patients, compared with normal mucosa. p53 and CD10 expression was only slightly increased in adenomas, but more frequently expressed in carcinomas. Bcl-2 expression was increased in adenomas, but decreased in carcinomas. *Conclusion*. This is the first study investigating collectively the expression of these molecules together in nonneoplastic mucosa, adenomas, and carcinomas from FAP patients. We find that beta-catenin and related proliferative and apoptotic factors (cyclin-D1, bcl-2, caspase-3, and Ki-67) are expressed early in the sequence, in adenomas. However, p53 and CD10 are often expressed later in the sequence, in carcinomas.

## 1. Introduction

Familial adenomatous polyposis (FAP) is the most common polyposis syndrome and affects 1 in 6850 to 1 in 29,000 live births [1]. FAP is caused by a germline mutation of the adenomatous polyposis coli (*APC*) gene [2]. Affected individuals develop up to several thousand adenomatous polyps in the colon and rectum. Furthermore, carcinoma invariably develops in FAP patients if the colon is not resected by the age of 40 or 50 [3].

The APC protein acts to bind beta-catenin, increasing beta-catenin turnover and decreasing its levels [4]. Beta-catenin in turn serves a dual purpose. It forms a complex with E-cadherin as well as other catenins at the cell membrane forming adherens junctions and mediates the interaction between the cadherin-catenin complex and the actin cytoskeleton. This interaction is vital for cell-cell adhesion. In addition, when Wnt ligands are present, binding of beta-catenin to APC is inhibited and beta-catenin levels are increased in the cytoplasm and nucleus. Beta-catenin acts together with TCF/LIF as a transcription factor for multiple genes, including *c-Myc*, *n-Myc, * and *cyclin-D1* [5]. Many *APC* mutations result in a truncated protein, with loss of the beta-catenin regulatory activity. This results in activation of the target genes of the Wnt signalling pathway, many of which are involved in cell cycle and proliferation.

APC mutations result in abnormal crypt proliferation in the gastrointestinal tract, resulting in adenoma formation [6]. Apart from causing multiple adenoma formation in FAP patients, it is also an early event in the adenoma-carcinoma sequence of sporadic tumours. However, the formation of invasive colorectal carcinomas requires further cumulative genetic changes, including the *RAS* family of genes, *SMAD4,* and *TP53.*

In this study, we investigated the expression of beta-catenin and downstream and related factors in nonneoplastic colonic mucosa, adenomas, and carcinomas from FAP patients, exploring the changes in expression along the adenoma-carcinoma sequence.

## 2. Methods

### 2.1. Case and Sample Selection

Sixteen patients with FAP who had developed colorectal adenocarcinoma were identified from the database of St. Mark's Hospital, London, UK. All patients had undergone colectomy at St. Mark's Hospital. Ethical approval for the study was obtained from Harrow Research Ethics Committee (reference number 3033).

The clinical history and histopathological reports and slides were reviewed. Multiple representative tissue blocks containing adenomas, adenocarcinomas and nonneoplastic mucosa were selected for each case. The material from the 16 patients included 152 adenomas (140 low grade dysplasia and 12 high grade dysplasia) and 19 adenocarcinomas (all moderately or poorly differentiated), including 2 separate synchronous carcinomas from 3 of the patients. There were 71 sections of nonneoplastic mucosa, of which 66 were adjacent to either adenomas or adenocarcinomas and 5 were away from tumours. 

### 2.2. Immunohistochemistry

The expressions of beta-catenin, p53, bcl-2, caspase-3, cyclin-D1, CD10, and Ki-67 were evaluated by immunohistochemistry using the avidin biotin immunodetection complex method. Two-micron-thick sections from formalin-fixed, paraffin embedded tissue were prepared, deparaffinised, and rehydrated. Endogenous peroxidase was blocked by incubation in hydrogen peroxide. Antigen retrieval was performed by microwaving in either ethylenediaminetetraacetic acid (EDTA) or citrate buffer. Sections were incubated with normal goat serum for 10 minutes and then with the primary antibody for 60 minutes at room temperature. The sources, dilutions, and antigen retrieval buffers and duration of microwaving for the primary antibodies are presented in Table 1. The sections were washed and then incubated with goat anti-mouse biotinylated immunoglobulin for 30 minutes, followed by streptavidin peroxidase for 30 minutes. The slides were developed in 3, 3′-diaminobenzidine (DAB), followed by a haematoxylin counterstain. Sections from nonneoplastic colonic mucosa from non-FAP patients, normal breast, and endometrium were used as positive controls, and for each case a section from which the primary antibody was replaced by phosphate buffered saline was used as a negative control.

### 2.3. Assessment of Expression

All sections were examined by light microscopy for the presence of expression and cellular distribution of the proteins (between the cell membrane, cytoplasm, and nucleus) in nonneoplastic mucosa, adenomas and adenocarcinomas. Cell staining intensity was scored as negative (0), weak (1+), moderate (2+), and strong (3+). Tumours showing antigen expression showed intratumoural heterogeneity in the intensity of staining. For each case, the percentage of cells with the predominant staining intensity was recorded. For statistical purposes, cases which were moderately (2+), or strongly (3+) positive in more than 10% of cells were designated overall positive, while cases in which expression was weak (1+) or seen in less than 10% of cells were considered overall negative. Slides were scored independently by two investigators (J. Wang and M.El-Bahrawy), and disagreements were resolved by review and a consensus was reached. 

### 2.4. Statistical Analysis

The presence of significant differences in expression of beta-catenin, p53, bcl-2, cyclin-D1, caspase-3, and CD10 between adenomas, carcinomas, and nonneoplastic mucosa was assessed by chi-square (*χ* ^2^) test. Ki-67 indices were compared between adenomas, carcinomas, and nonneoplastic mucosa by using the Mann-Whitney *U* test, with a *Z* score of >1.96 or <−1.96 significant at *P* < 0.05. A different statistical test was used for the Ki-67 indices, as Ki-67 were scored as percentages of cells, rather than assigned overall positive or negative scores. Therefore a nonparametric numerical statistical analysis, instead of a categorical one, was used.

All statistical analyses were performed using the statistical functions in Microsoft Excel.

## 3. Results

### 3.1. Histology

The adenomas comprised 140 adenomas with low grade (mild or moderate) dysplasia and 12 adenomas with high grade dysplasia. The carcinomas comprised 19 moderately differentiated and poorly differentiated adenocarcinomas. For comparison, 71 sections of nonneoplastic mucosa were analysed. Of these, 66 sections were adjacent to either adenomas or adenocarcinomas, while 5 were sections away from tumours.

### 3.2. Expression of Markers in Nonneoplastic Epithelium

**Figure 1:**
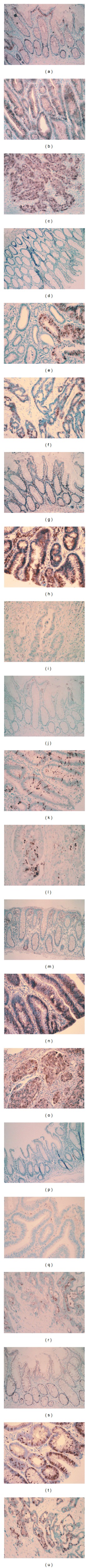
Immunohistochemical stained microphotographs of sections staining for beta-catenin in nonneoplastic mucosa (a), adenoma (b), and adenocarcinoma (c); Ki-67 in nonneoplastic mucosa (d), adenoma (e), and adenocarcinoma (f); p53 in non-neoplastic mucosa (g), adenoma (h), and adenocarcinoma (i); bcl-2 in nonneoplastic mucosa (j), adenoma (k), and adenocarcinoma (l); cyclin-D1 in nonneoplastic mucosa (m), adenoma (n), and adenocarcinoma (o); caspase-3 in nonneoplastic mucosa (p), adenoma (q), and adenocarcinoma (r); CD10 in nonneoplastic mucosa (s), adenoma (t), and adenocarcinoma (u). (a, d, g, j, m, p, and s at 100x magnification; b, c, e, f, h, i, k, l, n, o, q, r, t, and u at 200x magnification).

Membranous and cytoplasmic staining for beta-catenin positivity was seen in all cases of nonneoplastic epithelium (100%, Figure 1(a)). The membranous and cytoplasmic staining was most pronounced in the surface mucosa. In contrast, nuclear positivity was only seen in 6 cases of nonneoplastic mucosa (9.0%). Where nuclear staining was present, it was located at the base of the crypts, presumably in stem cells. No difference in staining was noted for nonneoplastic epithelium adjacent to or away from tumours.

Staining for p53 was not seen in any of the sections of nonneoplastic epithelium (Figure 1(d)). Bcl-2 staining was only seen in one section (1.4%), where positivity was seen in the cytoplasm and nuclei (Figure 1(g)). Cyclin-D1 was present in 3 cases (4.5%), with staining predominantly in the nuclei, with some staining in the cytoplasm (Figure 1(j)). Caspase-3 staining was not seen in nonneoplastic epithelium (Figure 1(m)). CD10 positivity was present in 4 cases (6.1%) and seen in the cell membranes (Figure 1(p)). The Ki-67 positivity in nonneoplastic epithelium was 5%, which was predominantly in cells in the basal one-third of the crypts (Figure 1(s)). 

### 3.3. Expression of Markers in Adenomas

In adenomas, membranous and cytoplasmic expression of beta-catenin was seen in all cases (100%, Figure 1(b)). However, there was also nuclear staining in 147 cases (99.3%). Compared with nonneoplastic epithelium, there was a significantly increased frequency of nuclear staining for both low and high grade adenomas combined (*χ* ^2^ > 25, *P* < 0.01). There was no significant difference in the frequency of nuclear staining between low grade and high grade lesions, 99.3% and 100%, respectively (*χ* ^2^ = 0.003, *P* = 0.99).

For p53, positive nuclear staining was seen in 9 low grade adenomas (7.1%) and in 3 high grade adenomas (25%). Compared with nonneoplastic epithelium, there was statistically significant increased frequency of staining for p53 in all low and high grade adenomas combined (*χ* ^2^ > 6.0, *P* = 0.014, Figure 1(e)). Bcl-2 staining was cytoplasmic and nuclear (Figure 1(h)) and seen in 68 low grade adenomas (48.6%) and 6 high grade adenomas (50%). Cyclin-D1 positivity was nuclear and cytoplasmic (Figure 1 (k)) and seen in 111 low grade adenomas (88.1%) and 11 high grade adenomas (91.7%). Caspase-3 staining was cytoplasmic (Figure 1(n)) and seen in 96 low grade adenomas (72.2%) and 6 high grade adenomas (50%). Compared with nonneoplastic mucosa, there was significantly increased staining for bcl-2, caspase-3, and cyclin-D1 in all adenomas combined (*χ* ^2^ > 25, *P* < 0.01). CD10 staining was rarely positive (Figure 1(q)), with only 1 low grade adenoma (0.8%) and 1 high grade adenoma (9.1%) staining in the cell membranes. CD10 expression frequency was not statistically different between nonneoplastic epithelium and adenomas (*χ* ^2^ = 3.41, *P* = 0.06). The Ki-67 index was 37% and 32% in the low grade and high grade adenomas, respectively (Figure 1(t)). There was no statistically significant difference in the Ki-67 index between low and high grade adenomas (*Z* = 0.966, *P* = not significant (NS)). However, the Ki-67 index for the adenomas combined was statistically increased compared with the nonneoplastic epithelium (*Z* = 11.6, *P* < 0.05).

### 3.4. Expression of Markers in Carcinomas

Beta-catenin staining was seen only in the cytoplasm and nuclei of carcinoma cells (Figure 1(c)), being positive in 14 cases (93.3%). Compared with adenomas, there was no statistically significant increase in frequency of nuclear staining (*χ* ^2^ = 0.03, *P* = 0.87).

For p53, positive nuclear staining was seen in 12 cases (70.6%, Figure 1(f)). Compared with adenomas, there was statistically significant increased frequency of staining for p53 in carcinomas (*χ* ^2^ = 23.3, *P* < 0.01). Bcl-2 staining was cytoplasmic and nuclear (Figure 1(i)) and seen in only 1 case (5.6%). There was a statistically significant decrease in staining frequency in carcinomas compared with adenomas for bcl-2 (*χ* ^2^ = 6.23, *P* = 0.012). Cyclin-D1 positivity was nuclear and cytoplasmic (Figure 1(l)) and seen in 13 cases (81.2%). No statistically significant difference in staining was seen between adenomas and carcinomas for cyclin-D1 (*χ* ^2^ = 0.046, *P* = 0.83). Caspase-3 staining was cytoplasmic (Figure 1(o)) and seen in 12 cases (75%). There was not a difference in staining frequency between adenomas and carcinomas for caspase-3 (*χ* ^2^ = 0.025, *P* = 0.87). CD10 staining was positive in the cell membranes in 6 cases (40%, Figure 1(r)). This was significantly increased compared with adenomas (*χ* ^2^ > 25, *P* < 0.01). The Ki-67 index was 41% (Figure 1(u)). The Ki-67 index for carcinomas was not statistically different from that of adenomas (*Z* = −1.24, *P* = *NS*), but increased compared with the nonneoplastic epithelium (*Z* = 6.20, *P* < 0.05).

Expression profiles of the different molecules in nonneoplastic mucosa, adenomas, and carcinomas are summarised in Table 2.

## 4. Discussion

The current results confirm our previous findings that while the expression of beta-catenin was confined predominantly to the cell membrane in nonneoplastic epithelium with weak staining in the cytoplasm, in adenomas and carcinomas, there was nuclear staining as well as increased cytoplasmic staining in more than 90% of cases [7]. In this current study, we show that this expression profile was present in both low grade and high grade adenomas as well as carcinomas, with similar percentages (93%–100%). This shows that nuclear localisation of beta-catenin is an early phenomenon in the adenoma-carcinoma sequence in FAP patients. This is expected due to the germline APC mutation in these patients. Furthermore, cytoplasmic and nuclear beta-catenin is also frequently seen in sporadic adenomas and carcinomas [8, 9]. There do not seem to be differences in nuclear positivity frequencies between sporadic and FAP tumours (both adenomas and carcinomas).

Mutations with subsequent accumulation of the mutated *TP53* gene product are well recognised as a late event in the adenoma-carcinoma sequence for sporadic tumours. However, for FAP patients, previous studies have shown *TP53* mutations occurring in adenomas [10]. Immunohistochemistry studies also showed that 37% of adenomas from FAP patients expressed p53, compared with 20% of sporadic adenomas [11]. In our study, we found that 7.1% of low grade adenomas expressed p53, compared with 25% of high grade adenomas and 70% of invasive carcinomas. These results suggest that although some p53 overexpression occurs in early lesions, the majority of the changes occur in the transformation from high grade dysplasia to invasive tumours. Although we did not compare our results directly with sporadic tumours, our results suggest that the incidence of p53 overexpression in FAP neoplasms is similar to that recognised for sporadic colorectal tumours, where 32% of adenomas and 67% of carcinomas expressed p53 [12].

The expression of bcl-2 has been studied extensively in sporadic colorectal tumours, but not in FAP patients. There has been only one study of bcl-2 in FAP patients, but only adenomas were analysed. In sporadic tumours, several studies have shown consistent results, with bcl-2 expressed in 80%–90% of adenomas, but the expression was reduced to 30%–50% in carcinomas [13, 14]. Our study shows 48.7% of adenomas being positive for bcl-2 but only 5.6% positivity in carcinomas. The difference in the percentages in comparison with sporadic tumours may be due to differences in scoring criteria, but may also be due to actual decrease in bcl-2 expression in FAP lesions. Nevertheless, there is a similar trend in the downregulation of the antiapoptotic protein with invasion seen in FAP patients, as for sporadic tumours, and this reflects similar apoptotic signalling changes in both FAP and sporadic tumours.

Caspase-3 is a protease that interacts with other caspases and is a key component of the executioner apoptotic pathway. In this study, we found that caspase-3 expression is absent in nonneoplastic epithelium, but positive in 72.2% of low grade adenomas, 50% of high grade adenomas and 75% of carcinomas. This is the first study to show caspase-3 expression along the adenoma-carcinoma sequence in FAP patients and to show that caspase-3 expression occurs early in the sequence, but persists into invasive carcinomas, unlike the expression of the antiapoptotic protein bcl-2. There have been few studies on caspase-3 in colorectal cancers in general. One study showed increased expression in adenomas compared with nonneoplastic mucosa, but decreased in carcinomas compared with adenomas [15]. However, it has also been shown that caspase-3 expression correlated with a higher risk of recurrence [16].

CD10 is a cell surface metalloprotein endopeptidase, involved in cleaving cytokines. It was initially discovered to be expressed in various lymphomas, but has subsequently been found widely expressed in various mesenchymal cells and mesenchymal-derived tumours. CD10 expression in sporadic colorectal tumours has been rarely studied, and to date there are no published data on CD10 in FAP patients. We found that CD10 is rarely expressed in nonneoplastic epithelium or adenomas (0.8%–9.1%). In contrast, 40% of carcinomas expressed CD10. These results are similar to data on sporadic tumours, with CD10 expression associated with an invasive phenotype rather than adenomas, with more than 50% of carcinomas expressing CD10 [17–19]. Furthermore, CD10 expression in colorectal cancers is associated with increased invasiveness, lymph node involvement, and liver metastasis [20, 21]. These results suggest that FAP tumours have similar mechanisms of acquiring invasive potential as sporadic tumours.

Since cyclin-D1 is a downstream target of beta-catenin, we expected cyclin-D1 expression to be increased in both adenomas and carcinomas. This was shown in our study, where 88.1% of low grade adenomas, 91.7% of high grade adenomas, and 81.2% of carcinomas were positive, compared with 4.5% of neoplastic epithelium. A previous study on FAP adenomas showed 40%(±20%) expression of cyclin-D1 in adenomas, compared with no expression in nonneoplastic mucosa [22]; however, no carcinomas were studied. In contrast, another study showed increased expression in sporadic carcinomas, but adenomas were not studied [23]. Nevertheless, these results show that increased cell proliferation may be linked to beta-catenin dysregulation.

Finally, while the normal Ki-67 proliferation index is 5% in nonneoplastic epithelium, with Ki-67 staining seen mostly in the base of crypts, the Ki-67 index is 37% in low grade adenomas, 32% in high grade adenomas, and 41% in invasive carcinomas; this early increase in cell proliferation mirrors the increased expression of beta-catenin and cyclin-D1. This is expected as these factors drive cell proliferation. This is seen also in sporadic adenomas and carcinomas, where the Ki-67 index was 30% and 38% in adenomas and carcinomas, respectively [12].

Overall, we find that beta-catenin and related proliferative factors (cyclin-D1 and Ki-67) are overexpressed in adenomas. Our data suggest that increased cell turnover is an early event in the FAP adenoma-carcinoma sequence, as the frequency of these factors is significantly increased in adenomas compared with nonneoplastic epithelium and further increased in carcinomas compared with adenomas. In contrast, changes in the apoptotic pathway appear to be more complex in the sequence of events. While the apoptotic effector protease caspase-3 is upregulated in adenomas and persists in carcinomas, the antiapoptotic regulator bcl-2 is upregulated in adenomas, but downregulated in invasive carcinomas. In contrast, p53 overexpression occurs more commonly later in the sequence, in invasive carcinomas. A recent study suggests that the Wnt signalling results in increased apoptosis via increased expression of the proapoptotic factors bok and bax [24]. However, Wnt signalling also induces antiapoptotic factors bcl-W and bcl-2 expression [25, 26]. Since both pro- and antiapoptotic factors are upregulated in adenomas, tumour progression is likely caused by a balance between these factors. Later, as tumours become invasive, it may be that p53 mutations occur which substitute for the effect of bcl-2 in counteracting the proapoptotic factors. Interestingly, several previous papers also have reported an inverse relationship in p53 and bcl-2 expression in adenomas and carcinomas [14, 27]. Furthermore, both p53 overexpression and loss of bcl-2 were associated with poorer prognoses [28, 29].

## 5. Conclusions

In conclusion, our study shows that the immunophenotypic changes in proliferation and apoptosis markers seen in FAP tumours are similar to those seen in sporadic tumours, suggesting similar tumour initiation and progression pathways. The results also suggest that disturbances in apoptosis are more influential factors in tumour progression in comparison to cell proliferation, which seems to be initiated in the early stages of tumourigenesis. We therefore recommend that these apoptotic pathways be the point of focus for further research and development of therapeutic targets and agents.

## Figures and Tables

**Table 1:** Primary antibody sources, dilution, and antigen retrieval buffers and conditions.

Antibody	Source	Dilution	Antigen retrieval buffer	Microwaving time
Beta-catenin	LEICA NCL-B-CAT	1 : 100	Citrate	30 min
p53	LEICA NCL-p53-D07	1 : 100	Citrate	20 min
Bcl-2	DAKO M0887	1 : 200	EDTA	30 min
Cyclin-D1	Thermo Shandon RM-9104-S	1 : 25	Citrate	40 min
Caspase-3	Cell Signalling CAT: 9664	1 : 800	EDTA	20 min
CD10	LEICA NCL-L-CD10-270	1 : 20	EDTA	30 min
Ki-67	LEICA NCL-L-KI67-MM1	1 : 100	Citrate	30 min

**Table 2:** Frequency of expression of beta-catenin, bcl-2, cyclin-D1, caspase-3, CD10, and Ki-67 in nonneoplastic mucosa, adenomas, and carcinomas.

	Beta-catenin	p53	Bcl-2	Cyclin-D1	Caspase-3	CD10	Ki-67 index
	(nuclear only)						
Nonneoplastic mucosa	6/67 (9.0%)	0/71 (0%)	1/71 (1.4%)	3/66 (4.5%)	0/68 (0%)	4/66 (6.1%)	5%
Low grade adenomas	135/136 (99.3%)	9/126 (7.1%)	68/140 (48.6%)	111/126 (88.1%)	96/133 (72.2%)	1/136 (0.8%)	37%
High grade adenomas	12/12 (100%)	3/12 (25%)	6/12 (50%)	11/12 (91.7%)	6/12 (50%)	1/11 (9.1%)	32%
Invasive carcinomas	14/15 (93.3%)	12/17 (70.6%)	1/18 (5.6%)	13/16 (81.2%)	12/16 (75%)	6/15 (40%)	41%
